# Proteome and Metabolome Analyses of Albino Bracts in *Davidia involucrata*

**DOI:** 10.3390/plants14040549

**Published:** 2025-02-11

**Authors:** Qinsong Liu, Jinqiu Wang, Yuying Li, Lei Xu, Wenjuan Xu, Ramesh R. Vetukuri, Xiao Xu

**Affiliations:** 1 Key Laboratory of Southwest China Wildlife Resources Conservation, China West Normal University, Ministry of Education, Nanchong 637009, China; yyl0215@163.com (Y.L.); xl2017128@163.com (L.X.); xuwj2006@163.com (W.X.); 2 Key Laboratory of Coarse Cereal Processing, Ministry of Agriculture and Rural Affairs, School of Food and Biological Engineering, Chengdu University, Chengdu 610106, China; wangjinqiu@cdu.edu.cn; 3 Department of Plant Breeding, Swedish University of Agricultural Sciences, SE-230 53 Alnarp, Sweden; ramesh.vetukuri@slu.se

**Keywords:** albino, chloroplast, photosynthesis, proteomics, metabolomics

## Abstract

Although the mechanisms underlying albino phenotypes have been examined in model plants and major crops, our knowledge of bract albinism is still in its infancy. *Davidia involucrata*, a relic plant called dove tree, is best known for the intriguing trait with a pair of white bracts covering the capitula. Here, comparative physiological, cytological, proteomic, and metabolomic analyses were performed to dissect the albinism mechanism of *D. involucrata* bracts. The bracts exhibited low chlorophyll and carotenoid contents, reduced photosynthetic efficiency, and impaired chloroplast structure. The severe deficiency of photosynthetic pigments and the substantial decrease in cuticle thickness made the bracts light-sensitive. In total, 1134 differentially expressed proteins (DEPs) were obtained between bracts and leaves. Pathway enrichment analysis of DEPs revealed that photosynthetic pigment biosynthesis and photosynthesis were suppressed, whereas protein processing in endoplasmic reticulum, flavonoid biosynthesis, and the ubiquitin–proteasome system (UPS) were activated in bracts. Strikingly, DEPs implicated in chloroplast development, including PPR and AARS proteins, were mainly down-regulated in bracts. We further investigated albinism-induced metabolic changes and detected 412 differentially abundant metabolites (DAMs). Among them, enhanced flavonoids accumulation can plausibly explain the role of bracts in pollinator attraction. Amino acids and their derivatives in bracts showed remarkably increased abundance, which might be causally linked to enhanced UPS function. Our work could lay foundations for understanding albinism mechanisms and adaptive significance of plant bracts and facilitate future utilization of *D. involucrata* resources.

## 1. Introduction

Plants with defects in chloroplast development or photosynthetic pigment metabolism usually exhibit albino, striped, variegated, etiolated, or pale-green leaves [1,2,3,4]. Among them, albino plants serve as potent tools for studying photosynthesis, chloroplast biogenesis and development, as well as chlorophyll (Chl) and carotenoid biosynthesis [1,3]. Furthermore, the albino trait holds great importance in ornamental horticulture due to its unique aesthetic appeal. The rarity and visual impact of albino plants make them a popular choice for landscaping [5]. Meanwhile, albino plants represent symbolic and cultural meanings in various societies globally, which further enhances their applications in ornamental horticulture. In addition, the increased accumulation of certain bioactive compounds in albino plants highlights their great nutritional and pharmaceutical potential [3,6].

Substantial progress has been achieved in dissecting gene regulatory networks underlying the albino phenotype, and multiple genes implicated in chloroplast development have been characterized [1,7]. Mounting evidence suggests that chloroplast development relies on the tightly coordinated action of chloroplast- and nuclear-encoded gene products [7]. These include nuclear-encoded pentatricopeptide repeat proteins (PPRs) involved in the modulation of chloroplast RNA metabolism [8] and aminoacyl-tRNA synthetases (AARSs) that regulate chloroplast ribosome biogenesis [9]. Loss-of-function mutations in genes encoding PPRs or AARSs resulted in albino phenotypes accompanied by chloroplast developmental disorders [9,10]. Notably, the highly conserved proteolytic machineries in eukaryotes, autophagy and the ubiquitin–proteasome system (UPS), are engaged in the turnover of chloroplast proteins to maintain normal organellar functions and manage stress responses [11,12]. In addition, silencing or mutations of Chl and carotenoid biosynthetic genes, such as *protochlorophyllide oxidoreductase* (*POR*), *phytoene desaturase* (*PDS*), and *ζ-carotene desaturase* (*ZDS*), can also lead to albinism [13,14,15]. Albino phenotypes differ among species and have been reported in model plants and crops, such as *Arabidopsis* [14], maize [15], tomato [16], rice [9], and cucumber [17]. However, the regulation of albinism in woody species is much less understood.

Due to lack of photosynthetic pigments, albino plants are often exposed to light stress, even when grown under normal light conditions [18]. Among various light stress responses in plants, the accumulation of ultraviolet (UV)-absorbing flavonoids is regarded as an effective strategy to alleviate photoinhibitory and photooxidative damage, mainly by their roles as sunscreens and reactive oxygen species (ROS) scavengers [19]. Indeed, a substantial number of flavonoids, in particular those with ortho-dihydroxylated B-rings, were shown to be considerably accumulated in albino plants [18,20]. Moreover, enhanced protein degradation and higher free amino acid contents were detected in albino leaves compared with green counterparts [21,22]. Apart from flavonoids and amino acids, other metabolites that were differentially accumulated in albinos included lipids, organic acids, carbohydrates, alkaloids, nucleotides and their derivatives, tannins, and phenolic acids [18,23]. Together, these previous studies unequivocally revealed that plant albinism is accompanied by metabolic reconfiguration and adaptation.

*Davidia involucrata* Baill., a Tertiary relict tree endemic to China, is the only extant species included in the family Davidiaceae and has horticultural, medicinal, and ecological importance [24,25]. Presently, natural *D. involucrata* populations are exclusively found in mountain forests of Southwestern and South–Central China [26]. Nevertheless, since the early 20th century, it has been introduced to various areas in Europe and North America as a popular ornamental plant in gardens and parks [27]. Its trivial name, “dove tree”, is derived from the beautiful and unique trait with a pair of white bracts covering the flowering heads (capitula), which resembles a dove in shape. Without sepals or petals, *D. involucrata* instead employs the bracts to protect pollen from rain and to facilitate pollinator attraction [28]. These bracts are small and green in the beginning, resembling leaves, but they undergo rapid growth and an albinism process as the flowers mature [28]. However, scarce information is available regarding the albinism mechanism as well as albinism-related physiological and metabolic features of *D. involucrata* bracts. Bracts are metamorphosed leaves and share the homologous origin with leaves [28]. In this study, quantitative proteomics and metabolomics were employed to simultaneously observe the differences at protein and metabolite levels between *D. involucrata* bracts and leaves. Furthermore, we compared their cellular ultrastructures, Chl and carotenoid contents, photosynthetic efficiency, membrane permeability, and antioxidant capacity. To the best of our knowledge, this is the first study systematically addressing the albinism phenomenon of *D. involucrata* bracts at multiple levels.

## 2. Results

### 2.1. Pigment Contents and Photosynthetic Characteristics in D. involucrata Bracts

**Figure 1:**
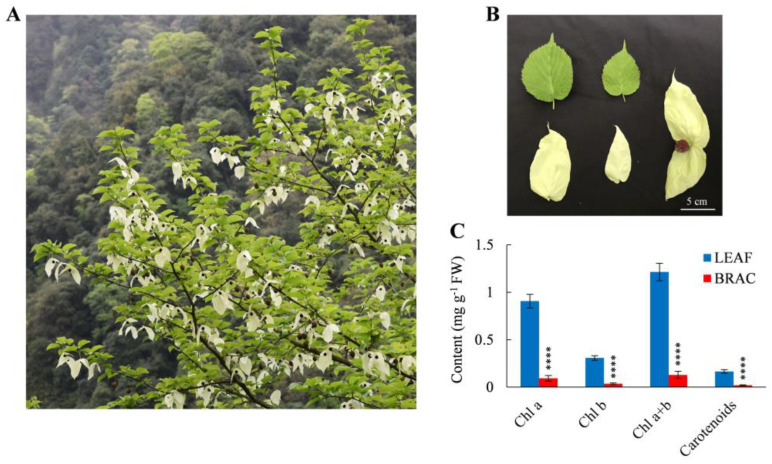
Phenotype and photosynthetic pigment contents of *D. involucrata* leaves and bracts. (**A**) Photo of *D. involucrata*. (**B**) Leaves, bracts, and flower of *D. involucrata*. (**C**) Contents of Chl a, Chl b, Chl a + b, and carotenoids in leaf samples (LEAF) and bract samples (BRAC). Values represent means ± SD (*n* = 4). Statistical significance (**** *p* < 0.0001) was revealed by Student’s *t*-test.

**Figure 2:**
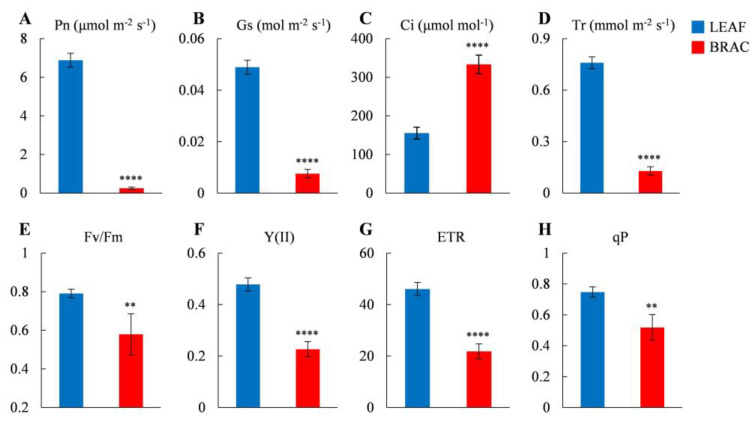
Photosynthetic characteristics of *D. involucrata* leaves and bracts. (**A**) Net photosynthetic rate (Pn). (**B**) Stomatal conductance (Gs). (**C**) Intercellular CO_2_ concentration (Ci). (**D**) Transpiration rate (Tr). (**E**) Maximum quantum yield of photosystem II (PSII) (Fv/Fm). (**F**) Effective quantum yield of PSII [Y(II)]. (**G**) Electron transport rate (ETR). (**H**) Photochemical quenching (qP). Values represent means ± SD (*n* = 4). Statistical significance (** *p* < 0.01, **** *p* < 0.0001) was revealed by Student’s *t*-test.

*D. involucrata* bracts and leaves were collected from flowering trees of the natural *D. involucrata* population (Figure 1A,B). We first compared their contents of Chl and carotenoid. The Chl a content in bracts was reduced by 89.67% relative to leaves (Figure 1C). A similar trend was detected for Chl b, Chl a + b, and carotenoid levels (Figure 1C). The observed deficiency of photosynthetic pigments agrees with the albino phenotype of *D. involucrata* bracts. Furthermore, gas-exchange measurements revealed that the net photosynthetic rate (Pn), stomatal conductance (Gs), and transpiration rate (Tr) in bracts decreased by 96.38%, 84.49%, and 82.95% compared to leaves, respectively, whereas intercellular CO_2_ concentration (Ci) in bracts increased by 114.38% (Figure 2A–D). Additionally, compared with leaves, bracts showed a substantial decrease in Chl fluorescence parameters, including the maximum quantum yield of photosystem II (PSII) (Fv/Fm), effective quantum yield of PSII [Y(II)], electron transport rate (ETR), and photochemical quenching (qP) (Figure 2E–H).

### 2.2. Ultrastructural Analysis of D. involucrata Bracts

**Figure 3:**
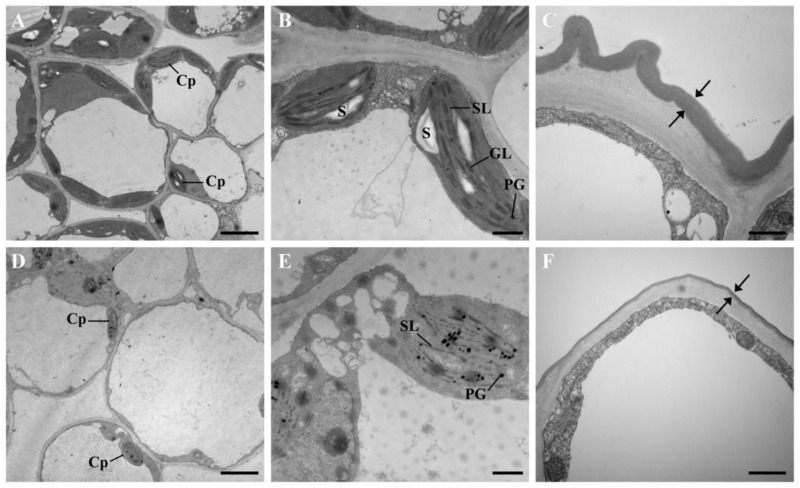
TEM analysis of *D. involucrata* leaves and bracts. (**A**–**C**) Electron micrographs of *D. involucrata* leaves. (**D**–**F**) Electron micrographs of *D. involucrata* bracts. Cp, chloroplast; S, starch granule; SL, stroma lamella; GL, grana lamella; PG, plastoglobule. Bars in (**A**,**D**), 5 μm; bars in (**B**,**C**,**E**,**F**), 1 μm.

We next performed ultrastructural studies of *D. involucrata* bracts and leaves using transmission electron microscopy (TEM). The number of chloroplasts in bracts was dramatically reduced in comparison to leaves (Figure 3A,D). Chloroplasts from leaves exhibited well-organized lamellar structures equipped with normally stacked grana, and contained relatively large starch granules as well as few plastoglobules (Figure 3B). By contrast, chloroplasts from bracts showed apparently reduced thylakoid system, the grana lamellae almost disappeared, the number of plastoglobules was substantially increased, and starch granules were hardly detectable (Figure 3E). These results suggest that albinism in *D. involucrata* bracts is closely associated with the observed abnormalities of chloroplasts. In addition, epidermis cell observation revealed that the cuticle in bracts was dramatically thinner compared to leaves (Figure 3C,F).

### 2.3. Screening and GO Classification of DEPs

**Figure 4:**
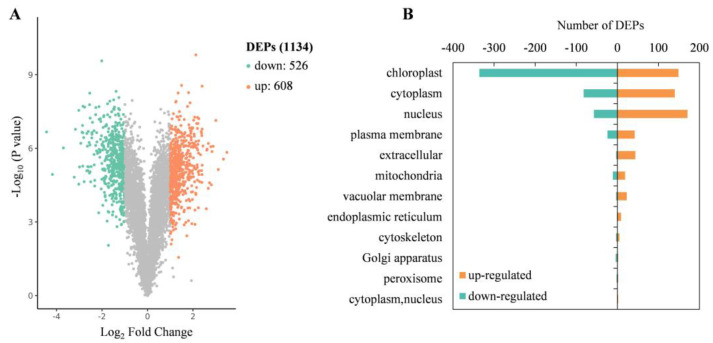
Proteomic alterations between *D. involucrata* bracts and leaves. (**A**) Volcano plot showing differential expression levels. Orange and green dots represent significantly up- and down-regulated DEPs, respectively. (**B**) Subcellular location of DEPs.

To identify proteins associated with albinism and investigate the molecular mechanisms underlying adaptation in *D. involucrata* bracts, comparative proteome analysis of bracts and leaves was conducted via the tandem mass tag (TMT)-based proteomic approach. Our analysis yielded a total of 285,895 spectra. After analyzing the spectra, 40,412 unique peptides were obtained. Ultimately, we identified 8524 proteins, from which 6832 were quantified (Table S1). Compared to leaves, 608 and 526 proteins were found to be up- and down-regulated in bracts, respectively (Figure 4A). These DEPs were also classified based on their subcellular localization. Among up-regulated proteins, 149 (24.51%) were localized to the chloroplast, 140 (23.03%) to the cytoplasm, and 171 (28.13%) to the nucleus (Figure 4B). By contrast, the localization of down-regulated proteins resulted in 336 (63.88%) to the chloroplast, 82 (15.59%) to the cytoplasm, and 57 (10.84%) to the nucleus (Figure 4B).

**Figure 5:**
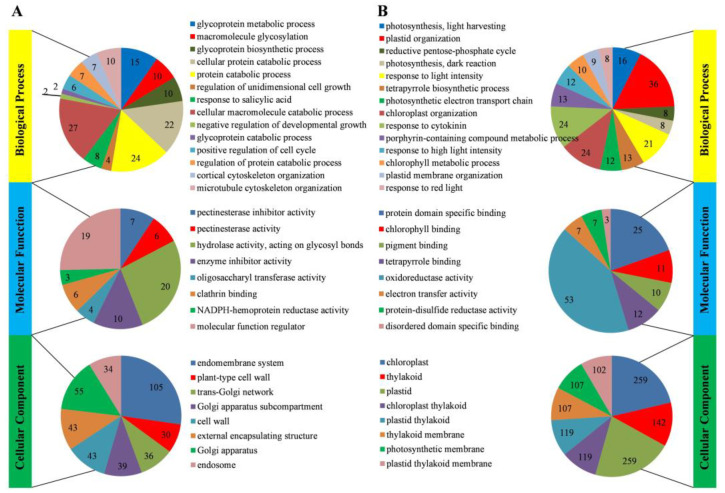
Go enrichment analysis of DEPs between *D. involucrata* bracts and leaves. (**A**) Distribution of up-regulated DEPs with GO annotation. (**B**) Distribution of down-regulated DEPs with GO annotation. The results were summarized in three categories, including biological process, molecular function, and cellular component. The number of DEPs in each GO term was displayed in a pie chart.

To inspect the functional classification of DEPs, we performed GO enrichment analysis. For up-regulated DEPs, the top three enriched GO terms in the biological process category were “cellular macromolecule catabolic process” (27 DEPs), “protein catabolic process” (24 DEPs), and “cellular protein catabolic process” (22 DEPs). In the category of molecular function, “hydrolase activity, acting on glycosyl bonds” (20 DEPs), “molecular function regulator” (19 DEPs), and “enzyme inhibitor activity” (10 DEPs) were the most abundant. In terms of cellular component, up-regulated DEPs were mainly associated with “endomembrane system” (105 DEPs), “Golgi apparatus” (55 DEPs), “cell wall” (43 DEPs), and “external encapsulating structure” (43 DEPs) (Figure 5A).

For down-regulated DEPs, the enriched GO terms in the biological process category were primarily associated with “plastid organization” (36 DEPs), “chloroplast organization” (24 DEPs), and “response to cytokinin” (24 DEPs). Within the molecular function category, down-regulated DEPs were mainly related to “oxidoreductase activity” (53 DEPs), “protein domain specific binding” (25 DEPs), and “tetrapyrrole binding” (12 DEPs). The enriched terms in the cellular component category included “chloroplast” (259 DEPs), “plastid” (259 DEPs), and “thylakoid” (142 DEPs) (Figure 5B).

### 2.4. KEGG Enrichment Analysis of DEPs

**Figure 6:**
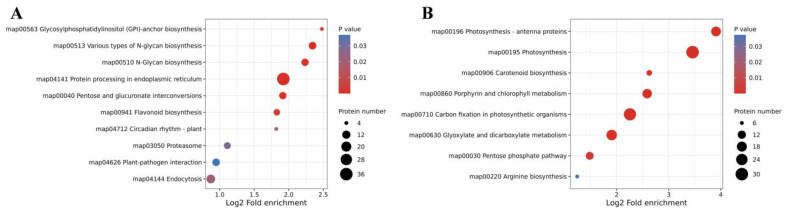
KEGG pathway classification for up-regulated DEPs (**A**) and down-regulated DEPs (**B**) between *D. involucrata* bracts and leaves.

We subsequently conducted KEGG enrichment analysis to address whether these DEPs could be implicated in specific pathways. For up-regulated DEPs, 10 significant pathways were enriched, including “protein processing in endoplasmic reticulum”, “proteasome”, and “flavonoid biosynthesis” (Figure 6A). Down-regulated DEPs were enriched in eight distinct pathways, including “photosynthesis”, “carbon fixation in photosynthetic organisms”, “photosynthesis—antenna proteins”, “porphyrin and chlorophyll metabolism”, and “carotenoid biosynthesis” (Figure 6B).

**Figure 7:**
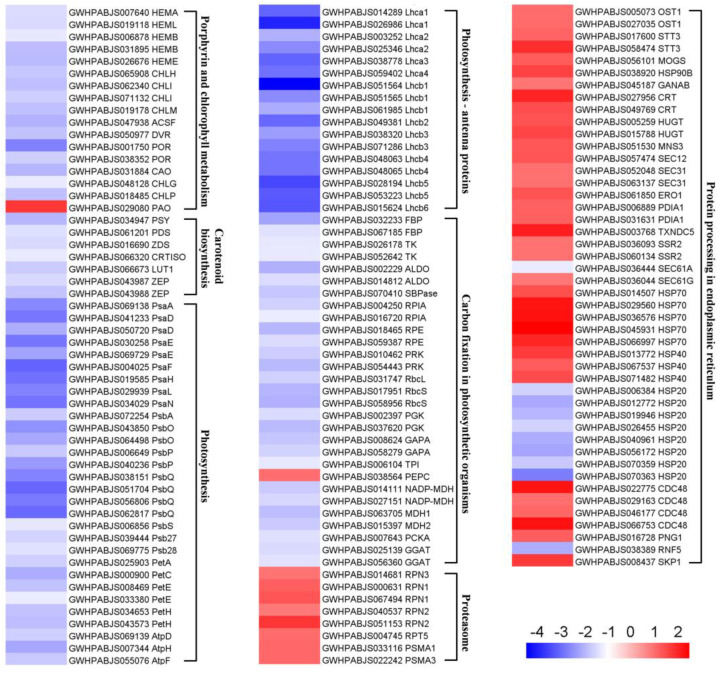
Heatmap of DEPs implicated in chlorophyll metabolism, carotenoid biosynthesis, photosynthetic metabolism, and protein quality control. Heatmap was generated based on the log_2_ fold change values (BRAC vs. LEAF). Detailed annotation information of these DEPs can be found in Table S2.

Based on KEGG enrichment analysis, we extracted the DEPs involved in several important pathways and visualized them on a heatmap (Figure 7; Table S2). Nearly all DEPs assigned to the “porphyrin and chlorophyll metabolism” pathway were suppressed, except for pheophorbide a oxygenase (PAO), which plays a pivotal role in chlorophyll breakdown [29]. All seven DEPs implicated in “carotenoid biosynthesis” were significantly down-regulated. There were 30 and 17 DEPs mapping to “photosynthesis” and “photosynthesis—antenna proteins”, respectively, and all these DEPs were suppressed. More specifically, 47 DEPs derived from these two pathways are involved in light reaction and functionally related to PSI (PsaA, PsaD, PsaE, PsaF, PsaH, PsaL, and PsaN), PSII (PsbA, PsbO, PsbP, PsbQ, PsbS, Psb27, and Psb28), cytochrome b6/f complex (PetA, PetC), photosynthetic electron transport (PetE, PetH), F-type ATPase (AtpD, AtpH, and AtpF), and light-harvesting chlorophyll protein complex (LHC) (Lhca1–4, Lhcb1–6). There were 29 DEPs associated with “carbon fixation in photosynthetic organisms”, with 28 decreased and 1 increased. All 8 members engaged in the “proteasome” pathway were substantially up-regulated. Additionally, a total of 46 DEPs were assigned to “protein processing in endoplasmic reticulum”, among which 36 and 10 members were up- and down-regulated, respectively.

### 2.5. Alterations in the Metabolomics of D. involucrata Bracts

**Figure 8:**
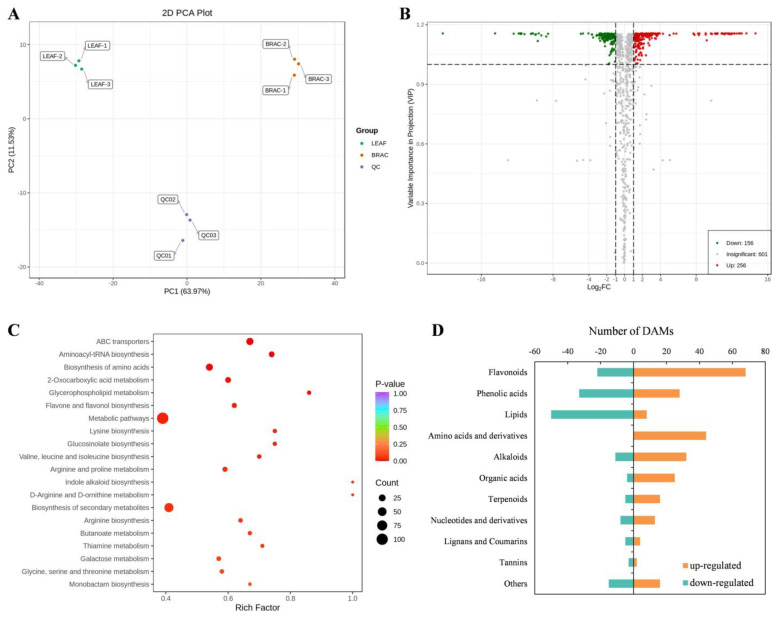
Analysis of DAMs between *D. involucrata* bracts and leaves. (**A**) PCA analysis. (**B**) Volcano plot of DAMs. Red and green dots represent significantly up- and down-regulated DAMs, respectively. (**C**) KEGG analysis of DAMs. (**D**) Classification of DAMs.

To further investigate albinism-induced metabolic changes, metabolite profiles in *D. involucrata* bracts and leaves were analyzed and quantitatively compared. In total, 1013 metabolites were identified. In the PCA model, PC1 and PC2 accounted for 63.97% and 11.53% of the total variance, respectively (Figure 8A). The PCA can clearly group these samples into distinct clusters, which points to significant metabolite differences between *D. involucrata* bracts and leaves. Consistent with PCA results, the OPLS-DA displayed a clear separation between these two groups (Figure S1). Moreover, R^2^X, R^2^Y, and Q^2^ were 0.811, 1, and 0.992, respectively, validating the reliability of OPLS-DA analysis.

Compared with leaves, there were 412 DAMs in bracts, of which 256 were up-regulated and 156 were down-regulated (Figure 8B; Table S3). KEGG analysis of DAMs revealed that many pathways in relation to amino acid metabolism were significantly enriched, including “biosynthesis of amino acids”, “lysine biosynthesis”, “valine, leucine and isoleucine biosynthesis”, “arginine and proline metabolism”, and “D-arginine and D-ornithine metabolism” (Figure 8C). Notably, the flavonoid biosynthesis-related pathway (i.e., flavone and flavonol biosynthesis) was also significantly enriched (Figure 8C). In addition, the classification with the largest number of DAMs was “flavonoids”, with 68 up-regulated metabolites and 22 down-regulated metabolites in bracts (Figure 8D). Importantly, 44 DAMs assigned to “amino acids and derivatives” were all up-regulated. Other metabolite classifications with an overall upward trend included “alkaloids” (32 up-regulated, 11 down-regulated), “organic acids” (25 up-regulated, 4 down-regulated), and “terpenoids” (16 up-regulated, 5 down-regulated). In contrast, the DAMs in the classification of “lipids” (8 up-regulated, 50 down-regulated) showed an overall downward trend. Together, these findings revealed the large-scale metabolic reprogramming associated with albinism in *D. involucrata* bracts.

### 2.6. Combined Proteomic and Metabolomic Analyses of Flavonoid Accumulation in D. involucrata Bracts

**Figure 9:**
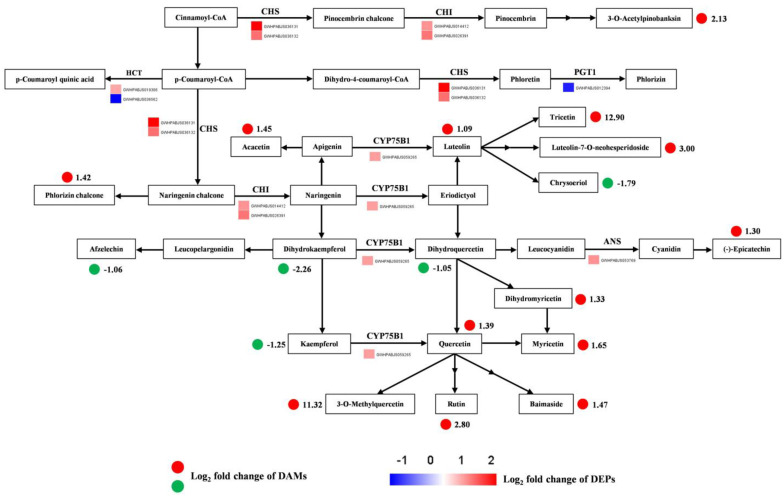
Integrated proteomic and metabolomic analyses of the flavonoid biosynthetic pathway. Red solid circles indicate up-regulation of DAMs, green solid circles indicate down-regulation of DAMs, and values on the right side represent the log_2_ fold change of DAMs (BRAC vs. LEAF). Heatmap represents the expression difference of DEPs and was generated based on the log_2_ fold change values (bracts vs. leaves). Detailed annotation information of these DEPs can be found in Table S4.

Modulation of flavonoid biosynthesis can serve as a pivotal adaptive mechanism of albino plants to environmental stress [3]. As shown in Figure 9, proteomic and metabolic profiles derived from *D. involucrata* bracts and leaves were exhibited in the flavonoid biosynthetic pathway. A total of 18 differentially accumulated flavonoids were found to be regulated by 9 DEPs in this pathway. The majority of the DEPs (seven out of nine) associated with flavonoid biosynthesis were up-regulated, including two chalcone synthases (CHSs), two chalcone isomerases (CHIs), one flavonoid 3′-monooxygenase (CYP75B1), and one anthocyanidin synthase (ANS) (Figure 9; Table S4). The metabolomic analysis revealed enhanced accumulation of four flavones (acacetin, luteolin, tricetin, and luteolin-7-O-neohesperidoside), one chalcone (phlorizin chalcone), two flavanonols (3-O-acetylpinobanksin and dihydromyricetin), five flavonols (quercetin, myricetin, 3-O-methylquercetin, rutin, and baimaside), and one flavanol (epicatechin) in *D. involucrata* bracts (Figure 9). To substantiate these findings, the levels of total flavonoids were also determined. In comparison to leaves, an approximate 1.5-fold increase in the total flavonoid contents was detected in bracts (Figure S2).

### 2.7. Evaluation of Antioxidant Status

**Figure 10:**
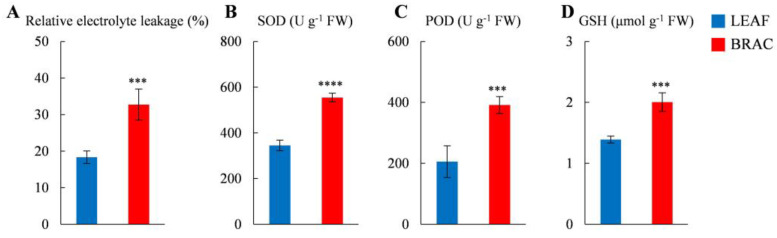
Relative electrolyte leakage (**A**), SOD activity (**B**), POD activity (**C**), and GSH content (**D**) in *D. involucrata* leaves and bracts. Values represent means ± SD (*n* = 4). Statistical significance (*** *p* < 0.001, **** *p* < 0.0001) was revealed by Student’s *t*-test.

We then examined whether *D. involucrata* bracts were subjected to light stress under normal light intensities. Strikingly, *D. involucrata* bracts exhibited significantly higher relative electrolyte leakage compared to leaves (Figure 10A), indicating increased membrane permeability in bracts. SOD and POD are known as crucial antioxidant enzymes in the redox regulation [30]. Enzyme assays demonstrated that SOD and POD activities in bracts were ~1.6- and ~1.9-fold greater than leaves, respectively (Figure 10B,C). Simultaneously, we detected augmented levels of the non-enzymatic antioxidant GSH in bracts (Figure 10D), which was also revealed by our metabolome analysis (Table S3). Collectively, these observations suggest that normal light levels are sufficient to trigger cellular damage and activate the antioxidant system in *D. involucrata* bracts.

## 3. Discussion

### 3.1. Albinism Might Relate to Chloroplast Abnormalities

In plant cells, chloroplasts are organelles responsible for conducting photosynthesis and also act as important sites for metabolism [1,7]. The albinism phenomenon generally reflects impaired chloroplast development and functioning [9,14]. Consistent with this notion, abnormal chloroplasts containing reduced thylakoid membranes and lacking grana stacks were observed in *D. involucrata* bracts (Figure 3). These structural defects can prevent chloroplasts from functioning properly during photosynthesis. Indeed, Pn, Gs, and Tr decreased while Ci increased in *D. involucrata* bracts compared with leaves (Figure 2A–D), suggesting that photosynthetic decline in bracts was not caused by stomatal limitation but rather by chloroplast abnormalities. Furthermore, photoinhibition of photosynthesis occurred in bracts, as evidenced by lower Fv/Fm, Y(II), ETR, and qP (Figure 2E–H). The aforementioned photosynthetic characteristics are similar to those seen in rice albino mutants [31].

Chloroplasts are known to harbor three membrane systems: the thylakoid membranes as well as the outer and inner envelope membranes [32]. The envelope of chloroplasts acts as their boundary membrane, which ensures the compartmentalization of metabolism between cytosol and chloroplasts [33,34]. The thylakoid membranes contain the major photosynthetic complexes (i.e., PSI, PSII, cyt b6/f complex, and ATPase), which consist of subunits that are encoded by either the nuclear or the chloroplast genome [35,36]. The biogenesis of these complexes, which is essential for the structure and function of thylakoids, relies on coordinated transport as well as assembly into high-molecular-weight structures [37,38]. Through diverse sorting mechanisms, nuclear-encoded thylakoid proteins are initially imported into chloroplasts and subsequently targeted to thylakoid membranes [39]. Notably, the formation of grana is governed by the supramolecular organization of photosynthetic complexes and their interactions [38,40]. In this study, all DEPs associated with PSI, PSII, LHC, cyt b6/f, photosynthetic electron transport, and ATPase were decreased (Figure 7; Table S2), thus leading to impaired thylakoid structure and inhibition of light reaction in bracts. Down-regulation of these proteins might be attributed to Chl limitation (Figure 1C), because the accumulation of photosynthetic complexes is determined by Chl synthesis and availability [41]. In addition, 28 out of 29 DEPs engaged in carbon fixation were repressed (Figure 7; Table S2), resulting in the disruption of this integral part of photosynthesis. A major rate-limiting step for photosynthesis is the CO_2_ fixation reaction catalyzed by Rubisco, which is constituted by eight large (RbcLs) and eight small subunits (RbcSs) [42]. We observed that one RbcL and two RbcSs displayed decreased expression in bracts. Sedoheptulose-1,7-bisphosphatase (SBPase) acts as a crucial Calvin cycle enzyme in controlling photosynthetic carbon assimilation [43]. Small reductions in SBPase activity were shown to significantly decrease photosynthetic efficiency [43,44], which agrees with the suppressed SBPase in *D. involucrata* bracts. Since the *SBPASE* mutation leads to inhibition of chloroplast biogenesis and starch accumulation [45], down-regulation of SBPase could plausibly explain the greatly reduced numbers of chloroplasts and starch granules in bracts.

Growing evidence indicates that PPRs act as major nuclear factors that modulate chloroplast gene expression and RNA metabolism [8,10,46]. Mutations in *PPR* genes were reported to cause aberrant chloroplast development and produce albino phenotypes [10,46]. The dysfunction of AARS genes, which play prominent roles in regulating plastidic ribosome biosynthesis, led to chloroplast developmental disorders and albinism in rice [9,47]. Here, we found that 13 out of 14 differentially expressed PPRs were suppressed and 5 AARSs were down-regulated (Table S5), suggesting that decreased accumulation of these proteins may account for impaired chloroplast structure in *D. involucrata* bracts.

### 3.2. Albinism Is Associated with Defective Photosynthetic Pigment Metabolism

Chloroplast biogenesis and development are generally accompanied by photosynthetic pigment accumulation [7]. Hence, albinos with abnormal chloroplasts often display defects in Chl and carotenoid accumulation [9,15,17]. Here, the contents of Chl a, Chl b, and carotenoids dramatically decreased in *D. involucrata* bracts (Figure 1C), thereby providing the most straightforward explanation for their albino phenotype. Consistent with reduced Chl contents, all 16 DEPs associated with Chl biosynthesis were substantially down-regulated in bracts (Figure 7; Table S2). Likewise, decreased accumulation of Chl biosynthetic enzymes was previously reported in a Chl-deficient mutant from *Brassica napus* [48]. Chl is subjected to degradation via a multiple-step process, referred to as the “PAO pathway”, acknowledging the importance of the enzymatic breakdown step catalyzed by PAO, which can provide structural basis for all downstream products of Chl breakdown [29]. Intriguingly, we found that bracts exhibited an approximate 3.7-fold increase in the protein abundance of PAO compared with leaves (Figure 7; Table S2). Therefore, apart from disruption of Chl biosynthesis, accelerated Chl degradation due to enhanced PAO accumulation may also contribute to a prominent reduction in Chl contents of bracts.

Decreased carotenoid levels coincide with down-regulation of carotenoid biosynthetic enzymes, including phytoene synthase (PSY), PDS, and ZDS (Figure 7; Table S2). PSY is known to catalyze the first and major flux-controlling step during carotenoid biosynthesis, and disruption of PSY isoforms resulted in an albino phenotype in tomato [49]. Loss of PDS or ZDS was reported to cause albinism by impairing Chl and carotenoid biosynthesis in *Arabidopsis* [14,50]. We therefore infer that albinism of *D. involucrata* bracts might also relate to reduced abundance of these carotenoid biosynthetic enzymes. The chloroplast signals its physiological and developmental status to the nucleus through a regulatory mechanism, termed retrograde signaling, which allows the expression of nuclear genes to be adjusted appropriately [51]. Besides being essential for carotenoid biosynthesis, ZDS has been implicated in retrograde signaling. Several nuclear genes that are regulated by retrograde signaling, including *Lhcb*, *RbcS*, *POR*, and *CAO*, were down-regulated in *zds* mutants [15,50]. Here, we observed that ZDS as well as 11 Lhcbs, two RbcSs, two PORs, and CAO simultaneously displayed reduced expression (Figure 7; Table S2), suggesting impaired retrograde signaling in *D. involucrata* bracts.

### 3.3. Albino Bracts Are Subjected to Light Stress

Severe deficiency of photosynthetic pigments readily exposes plants to light stress, which causes photooxidative damage, even under normal light intensities [18,20]. In this study, a series of analyses support the notion that *D. involucrata* bracts were exposed to light stress during the albinism process. First, our ultrastructural analysis revealed remarkably increased numbers of plastoglobules in chloroplasts of *D. involucrata* bracts (Figure 3B,E), indicating high oxidative stress in the photosynthetic apparatus [52]. Second, relative electrolyte leakage, which can reflect the degree of cell membrane damage under stress conditions [53], was greatly elevated in bracts compared to leaves (Figure 10A). Meanwhile, bracts exhibited higher SOD and POD activities as well as increased GSH levels compared to leaves (Figure 10B–D; Table S3), further suggesting the induction of antioxidant defense against oxidative damage in bracts. Third, the abundance of two zeaxanthin epoxidase (ZEP) proteins was significantly decreased in bracts compared to leaves (Figure 7; Table S2). The xanthophyll zeaxanthin is known to serve central photoprotective functions in plants [54]. ZEP catalyzes the epoxidation of zeaxanthin to violaxanthin in the xanthophyll cycle, and ZEP protein undergoes degradation upon high light exposure to ensure that high zeaxanthin levels are retained [54]. Thus, degradation of ZEP proteins might represent an adaptive strategy in light-stressed bracts. Fourth, most of the DEPs associated with protein processing in ER were induced in bracts (Figure 7; Table S2). Under adverse environmental conditions, the limited capability of the endoplasmic reticulum (ER) to process proteins usually results in massive accumulation of misfolded/unfolded proteins, which is detrimental to cell function [24]. Hence, we infer that the efficiency of protein processing might be promoted in bracts to cope with light stress. Heat shock proteins (HSPs) act as molecular chaperones required for the maintenance of protein homeostasis and play essential roles in stress management [24,55]. As expected, one HSP90B, five HSP70s, and three HSP40s assigned to protein processing in ER pathway were up-regulated in bracts. It might appear surprising that eight HSP20s, which are small HSPs (sHSPs), showed reduced expression in bracts. In fact, overexpression of *sHSPs* (i.e., *AsHSP17* or *AsHSP26.8*) from *Agrostis stolonifera* was reported to reduce plant heat and salt tolerance [56,57], pointing to the negative impact of certain *sHSPs* on abiotic stress response. Therefore, differential regulation of distinct HSPs in bracts may be critical for light stress adaptation. Lastly, *D. involucrata* bracts exhibited dramatically decreased cuticle thickness (Figure 3C,F). At the interface between plants and their environments, the thickened cuticle can function as a protective layer against excess UV or sunlight irradiance [58]. Hence, decreased cuticle thickness in *D. involucrata* bracts could explain (at least partially) their vulnerability to light stress. Consistent with our findings, a thinner cuticle was also observed in albino *Cephalanthera damasonium* individuals compared to green counterparts [59]. Collectively, the substantial decrease in photosynthetic pigment contents and cuticle thickness led to increased susceptibility of bracts to light stress, which triggered adaptive responses including degradation of ZEP proteins as well as activation of protein processing in ER and antioxidant defense.

### 3.4. Flavonoid Metabolism Is a Crucial Adaptive Trait for Bracts

Flavonoids can act as photoprotective molecules due to their UV-absorbing characteristics and antioxidant properties [19]. In this study, most of the differentially accumulated flavonoids were up-regulated in *D. involucrata* bracts (Figure 8D), which agrees with the observation in light-sensitive albino tea mutants [23]. Moreover, bracts showed higher total flavonoid contents than leaves (Figure S2), thereby serving as another adaptive mechanism against light stress, particularly UV-B exposure. Accordingly, majority of the DEPs implicated in the flavonoid biosynthetic pathway were substantially induced in bracts, including two CHSs, two CHIs, one CYP75B1, and one ANS (Figure 9; Table S4). Hence, the up-regulation of these proteins, which are well known as important players in flavonoid biosynthesis [60], provides molecular support for enhanced flavonoids accumulation in bracts. UV-B radiation was previously shown to increase the ratio of quercetin (effective antioxidant) to kaempferol (poor antioxidant) [61,62]. In the present study, quercetin and its derivatives (e.g., 3-O-methylquercetin, rutin, and baimaside) were up-regulated whereas kaempferol was down-regulated in bracts (Figure 9), suggesting that quercetin and its derivatives may play prominent roles in bract responses to light stress. Likewise, flavonoids with ortho-dihydroxylated B-rings (e.g., quercetin and its glycosides) were shown to serve photoprotective functions and preferentially accumulate in light-sensitive albino tea plants [20].

Besides providing protection against light stress, flavonoids are also known as major attractors for pollinators [63]. Pollen-collecting bees were found to be important pollinators of *D. involucrata*, and they prefer visiting capitula with white bracts rather than capitula with green ones [28]. Our study suggested that UV-absorbing flavonoids represent the main pigments in *D. involucrata* bracts during the albinism process, thus promoting pollination by attracting bees. Similar effects have been recently seen in glasshouse bracts of *Rheum nobile* [64].

### 3.5. Activation of the UPS in Albino Bracts

According to our metabolome data, amino acids and their derivatives in *D. involucrata* bracts exhibited increased abundance compared to leaves (Figure 8D; Table S3). Notably, higher levels of amino acids observed in albinos are often attributed to enhanced protein degradation [21,22]. In eukaryotic cells, autophagy and the UPS form the principle protein degradation machineries [65]. Vacuoles act as terminal lytic compartments in the plant endomembrane system to degrade cellular cargo [66], and autophagy is a vacuolar degradation pathway mediated by autophagy-related (ATG) proteins [11,25]. Here we detected neither altered abundance of ATG proteins nor increased formation of autophagic structures in *D. involucrata* bracts (Figure 3), implying that autophagic activity of bracts may not be elevated during the albinism process. The UPS is known to post-translationally modify cellular proteins with the ubiquitin molecule, ultimately leading to their degradation by the proteasome [67]. Our KEGG analysis of DEPs revealed that the “proteasome” pathway was significantly activated (Figure 6A; Table S2), and four DEPs implicated in the “ubiquitin mediated proteolysis” pathway were up-regulated in bracts (Table S6). As a ubiquitin-dependent segregase, cell division cycle 48 (CDC48) plays a critical role in the UPS [12]. Indeed, up-regulation of four CDC48 proteins was also detected in bracts (Figure 7; Table S2). Together, these results point to activation of the UPS in *D. involucrata* bracts during the albinism process, which could explain the enhanced amino acids accumulation. Intriguingly, upon oxidative stress, CDC48 was shown to be induced and mediate the turnover of ubiquitinated chloroplast proteins (e.g., RbcL, AtpB) via the UPS [12]. Hence, the decreased abundance of chloroplast proteins observed in bracts (Figure 7; Table S2) might relate to enhanced UPS function under light stress, which results in impaired chloroplast structure. Since amino acids are engaged in abiotic stress tolerance and are precursors of photoprotective metabolites [68], increased accumulation of amino acids in bracts due to activation of the UPS may also be crucial for adapting to light stress.

### 3.6. Possible Mechanisms of Albinism Induction in Bracts

Although our study provides valuable insights into the metabolic and proteomic changes linked to bract albinism, the exact triggers behind this phenomenon remain unclear. Based on previous research, we propose several potential mechanisms of albinism induction in *D. involucrata* bracts. First, phytohormones could be important players in the regulation of this process. Support for the hypothesis comes from the functional characterization of the transcription factor gene *abscisic acid, stress and ripening* (*DiASR1*) from *D. involucrata*. Ectopic expression of *DiASR1*, which is transcriptionally regulated by abscisic acid and gibberellin, induces an albino phenotype in transgenic *Arabidopsis* plants [69]. Impairment of auxin homeostasis has also been proposed to disrupt chloroplast development and cause the albinism phenomenon [70,71]. Second, overproduction of ROS may be responsible for albinism induction. In agreement with this notion, a series of genetic analyses previously demonstrated that superoxide anion radical (O_2_^•−^) over-accumulation caused the albino phenotype in *ospus1-1*, which is a rice mutant of chloroplast-localized pseudouridine synthase [72]. Third, disruption of mineral nutrition function might lead to albinism. Deficiency of certain mineral nutrients, such as iron or magnesium, can cause leaf chlorosis by impairing chlorophyll accumulation [73,74]. Indeed, albinism in orchids is associated with impaired mineral nutrition function, as evidenced by inhibition of mineral nitrogen assimilation and suppressed expression of transporters engaged in mineral nutrient translocation [21]. Lastly, the induction of albinism could be governed by epigenetic mechanisms. It has been shown that the development of the albino phenotype in *Agave angustifolia* plantlets may involve epigenetic regulation [75,76]. Hypermethylation at the promoter region of *OsAK1* was also reported to cause albinism in rice [77]. Further studies focusing on the aforementioned aspects could facilitate our understanding of the exact causes and biological basis of bract albinism in *D. involucrata*.

## 4. Materials and Methods

### 4.1. Plant Materials

Fresh leaves and bracts of *D. involucrata* were separately collected from flowering trees of the natural *D. involucrata* population (Figure 1A) located at Pingwu County in Sichuan, Southwest China on 11 April 2022. This *D. involucrata* population (104°32′ E, 32°19′ N, 1600 m altitude) has been described by our previous study [78]. At this stage, the color of bracts shows light yellow, bracts are turning white, and stamens begin to extend (Figure 1B), indicating that the stage of sampling is a turning point during the albinism process of *D. involucrata* bracts. The leaf samples were labeled as LEAF, and the bract samples were labeled as BRAC. Three biological replicates were used for proteomic and metabolomic analyses, and four biological replicates were used for the determination of physiological indices. After collection, leaf and bract samples were immediately frozen in liquid nitrogen and stored at −80 °C until analyzed.

### 4.2. Measurement of Physiological Indices

Relative electrolyte leakage and photosynthetic pigment contents were detected as previously described [79,80]. Gas exchange and Chl fluorescence parameters were measured with LI-6400 portable photosynthesis system (LI-COR, Bourne, MA, USA) and MINI-PAM-II photosynthesis yield analyzer (WALZ, Effeltrich, Germany), respectively. The contents of glutathione (GSH) and total flavonoids as well as superoxide dismutase (SOD) and peroxidase (POD) activities were determined using corresponding reagent kits according to the manufacturer’s protocol (Suzhou Comin, Suzhou, China).

### 4.3. Transmission Electron Microscopy

Small pieces (~1 × 2 mm) of *D. involucrata* leaves and bracts were excised and fixed with 2.5% glutaraldehyde. The specimens were subsequently post-fixed in 1% OsO_4_, dehydrated through an acetone series, and embedded in Epon 812. Ultrathin sections were prepared using a Leica UC7 microtome (Wetzlar, Germany). Sections of *D. involucrata* leaves and bracts were inspected with a JEM-1400Flash transmission electron microscope (JEOL, Tokyo, Japan).

### 4.4. TMT-Based Quantitative Proteomic Analysis

Extraction and digestion of the total proteins from *D. involucrata* leaves and bracts were performed according to the previous studies [81,82]. After trypsin digestion, the peptide segments were desalted with a Strata X C18 SPE column (Phenomenex, Torrance, CA, USA) and subsequently vacuum-dried. Peptides were reconstituted in 0.5 M TEAB and labeled by using the TMT kit (Thermo Fisher Scientific, Waltham, MA, USA). After thawing, the labeling reagents were dissolved in acetonitrile and mixed with peptides, followed by 2-h incubation at room temperature. The resulting labeled peptide mixtures were pooled, desalted, and then dried through vacuum centrifugation. Peptide fractionation was conducted via high pH reverse-phase HPLC using Agilent 300Extend C18 column (Agilent, Santa Clara, CA, USA). Finally, proteome analysis for fractionated peptides was performed as described using an LC-MS/MS system [83].

### 4.5. Database Search and Bioinformatics Analysis

Maxquant search engine (v.1.6.15.0) was subsequently employed to process the resulting MS/MS data. Tandem mass spectra were searched against *D. involucrata* protein sequences derived from annotation of whole genome assembly [84]. The following parameters were specified in database search: up to two missing cleavages were allowed using trypsin as the cleavage enzyme; for precursor ions, the mass tolerance in first search and main search was set to 20 ppm and 5 ppm, respectively; for fragment ions, the mass tolerance was 0.02 Da; carbamidomethyl on cysteine was considered as fixed modification; protein N-terminal acetylation, oxidation on methionine, and deamidation were considered as variable modifications. The FDR threshold was set to 1% for protein identification. TMT-6plex was selected as the quantitative method, and the *D. involucrata* proteins with a fold change ≥ 2 or ≤0.5 (BRAC vs. LEAF) and a *p*-value < 0.05 were regarded as differentially expressed proteins (DEPs). GO annotations of the *D. involucrata* proteome were conducted using the UniProt-GOA database (http://www.ebi.ac.uk/GOA/, accessed on 15 January 2025). The KEGG database was employed to annotate the protein pathway (http://www.genome.jp/kegg/, accessed on 15 January 2025). WoLF PSORT was utilized to predict the subcellular localization of *D. involucrata* proteins (http://www.genscript.com/psort/wolf_psort.html, accessed on 15 January 2025).

### 4.6. Metabolite Extraction and Profiling

The samples of *D. involucrata* leaves and bracts were freeze-dried and then ground using a mixer mill for metabolite extraction. The sample powder (50 mg) was extracted with 70% methanol solution (1.2 mL) overnight at 4 °C, followed by centrifugation at 12,000 rpm for 3 min. Prior to UPLC-MS/MS analysis, supernatants were separately collected and subjected to micropore membrane filtration (0.22 μm). The analyses of the resulting sample extracts were subsequently conducted by UPLC-ESI-MS/MS (UPLC, SHIMADZU Nexera X2, https://www.shimadzu.com.cn/; MS, Applied Biosystems 4500 Q TRAP, https://www.thermofisher.cn/cn/zh/home/brands/applied-biosystems.html, accessed on 15 January 2025) according to the previous study [85]. Qualitative and quantitative analyses of metabolites were performed by using MWDB (MetWare database, http://www.metware.cn/) and MRM (multiple reaction monitoring) according to the standard metabolic operating procedures, respectively [86].

### 4.7. Analysis of Metabolomics Data

The mass spectrum data were processed with Analyst 1.6.3 software. Statistics function prcomp within R 4.3.3 software was employed to perform unsupervised principal component analysis (PCA). R package (MetaboAnalystR) was utilized to conduct orthogonal partial least squares discriminant analysis (OPLS-DA). Differentially abundant metabolites (DAMs) between *D. involucrata* bracts and leaves were screened with variable importance in projection (VIP) ≥ 1 and |log_2_(fold change)| ≥ 1. In addition, the KEGG database was used to acquire metabolic pathway information.

## 5. Conclusions

**Figure 11:**
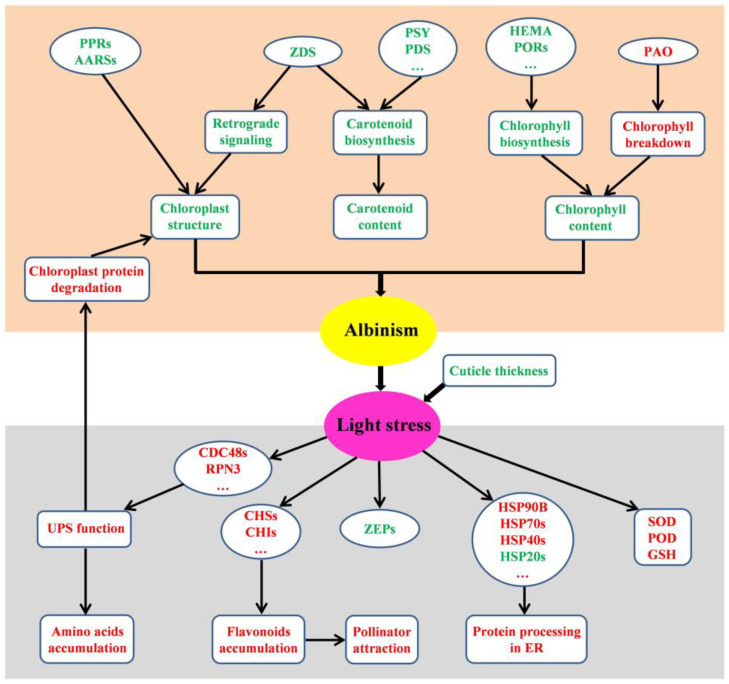
Schematic overview of potential albinism mechanism as well as metabolic and molecular adaptation in *D. involucrata* bracts. Red text represents up-regulation or enhancement, while green text represents down-regulation or impairment.

In summary, we revealed a cascade of physiological, molecular, and metabolic events in *D. involucrata* bracts during the albinism process (Figure 11). The DEPs associated with defects in photosynthesis, chloroplast development, and photosynthetic pigments accumulation represent the primary molecular mechanisms contributing to albinism. The albino bracts with decreased cuticle thickness are subjected to light stress. The secondary effects, such as degradation of ZEP proteins and activation of several biological processes (i.e, flavonoid biosynthesis, the UPS, protein processing in ER, and antioxidant defense), reflect the adaptive responses of *D. involucrata* bracts to light stress. Our work sheds light on the albinism and adaptation mechanisms of *D. involucrata* bracts and provides valuable information for further functional characterization of candidate genes. Based on our findings, it is crucial to provide optimal growing conditions (e.g., protection from harsh sunlight) for *D. involucrata* cultivation, particularly during anthesis. Furthermore, *D. involucrata* bracts contain high amounts of various bioactive compounds (e.g., flavonoids, GSH, alkaloids), and thus possess significant potential for pharmaceutical applications. A large number of DEPs identified in bracts indicate the reprogramming of gene networks, which could be regulated by epigenetic mechanisms such as DNA methylation and histone modifications. Comprehensive epigenetic analysis using samples collected from multiple developmental stages would be useful for elucidating the developmental dynamics of bract formation and albinism in future studies.

## Data Availability

The data presented in this study are available in the text and Supplementary Materials.

## Supplementary Materials

The following supporting information can be downloaded at: https://www.mdpi.com/article/10.3390/plants14040549/s1, Figure S1: Score plot generated from OPLS-DA of metabolic profiles in *D. involucrata* leaves and bracts; Figure S2: Total flavonoid content in *D. involucrata* leaves and bracts; Table S1: MS/MS spectrum database search analysis summary; Table S2: Details of DEPs associated with seven KEGG pathways; Table S3: Detailed information of DAMs; Table S4: Details of DEPs associated with flavonoid biosynthesis; Table S5: Details of DEPs associated with chloroplast development; Table S6: Details of DEPs implicated in ubiquitin mediated proteolysis.
