# Supplementary material for: Proteome and Metabolome Analyses of Albino Bracts in Davidia involucrata
Source: Plants (Basel). 2025 Feb 11;14(4):549. doi: 10.3390/plants14040549 (PMC11858999; doi:10.3390/plants14040549)
Supplement: Supplementary file 1 [file plants-14-00549-s001.zip › Supplementary Files/Supplementary Figures.pdf]

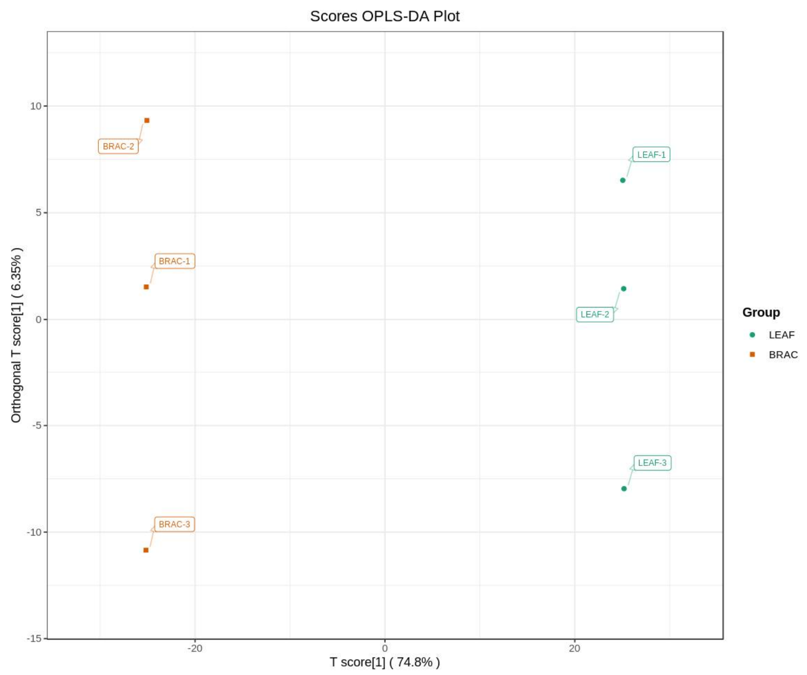

Figure S1. Score plot generated from OPLS-DA of metabolic profiles in *D. involucrata* leaves and bracts.

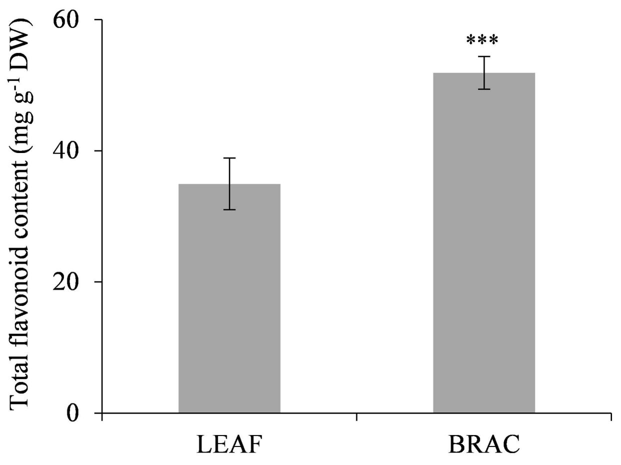

Figure S2. Total flavonoid content in *D. involucrata* leaves and bracts. Values represent means ± SD ($n = 4$). Statistical significance (***$P < 0.001$) was revealed by Student's *t* test.
